# Engagement With COVID-19 Public Health Measures in the United States: A Cross-sectional Social Media Analysis from June to November 2020

**DOI:** 10.2196/26655

**Published:** 2021-06-21

**Authors:** Daisy Massey, Chenxi Huang, Yuan Lu, Alina Cohen, Yahel Oren, Tali Moed, Pini Matzner, Shiwani Mahajan, César Caraballo, Navin Kumar, Yuchen Xue, Qinglan Ding, Rachel Dreyer, Brita Roy, Harlan Krumholz

**Affiliations:** 1 Center for Outcomes Research and Evaluation Yale New Haven Hospital New Haven, CT United States; 2 Section of Cardiovascular Medicine Department of Internal Medicine Yale School of Medicine New Haven, CT United States; 3 Signals Analytics New York, NY United States; 4 Department of Sociology Yale University New Haven, CT United States; 5 Foundation for a Smoke-Free World New York, NY United States; 6 College of Health and Human Sciences Purdue University West Lafayette, IN United States; 7 Department of Emergency Medicine Yale School of Medicine New Haven, CT United States; 8 Department of Chronic Disease Epidemiology Yale School of Public Health New Haven, CT United States; 9 Department of Medicine Yale School of Medicine New Haven, CT United States; 10 Department of Health Policy and Management Yale School of Public Health New Haven, CT United States

**Keywords:** COVID-19, public perception, social media, infodemiology, infoveillance, infodemic, social media research, social listening, social media analysis, natural language processing, Reddit data, Facebook data, COVID-19 public health measures, public health, surveillance, engagement, United States, cross-sectional, Reddit, Facebook, behavior, perception, NLP

## Abstract

**Background:**

COVID-19 has continued to spread in the United States and globally. Closely monitoring public engagement and perceptions of COVID-19 and preventive measures using social media data could provide important information for understanding the progress of current interventions and planning future programs.

**Objective:**

The aim of this study is to measure the public’s behaviors and perceptions regarding COVID-19 and its effects on daily life during 5 months of the pandemic.

**Methods:**

Natural language processing (NLP) algorithms were used to identify COVID-19–related and unrelated topics in over 300 million online data sources from June 15 to November 15, 2020. Posts in the sample were geotagged by NetBase, a third-party data provider, and sensitivity and positive predictive value were both calculated to validate the classification of posts. Each post may have included discussion of multiple topics. The prevalence of discussion regarding these topics was measured over this time period and compared to daily case rates in the United States.

**Results:**

The final sample size included 9,065,733 posts, 70% of which were sourced from the United States. In October and November, discussion including mentions of COVID-19 and related health behaviors did not increase as it had from June to September, despite an increase in COVID-19 daily cases in the United States beginning in October. Additionally, discussion was more focused on daily life topics (n=6,210,255, 69%), compared with COVID-19 in general (n=3,390,139, 37%) and COVID-19 public health measures (n=1,836,200, 20%).

**Conclusions:**

There was a decline in COVID-19–related social media discussion sourced mainly from the United States, even as COVID-19 cases in the United States increased to the highest rate since the beginning of the pandemic. Targeted public health messaging may be needed to ensure engagement in public health prevention measures as global vaccination efforts continue.

## Introduction

As COVID-19 continues its spread in the United States, a key to controlling the spread while vaccination efforts continue is to enlist the public in risk-mitigation behaviors [1,2]. Studying the public’s social media posts regarding COVID-19 public health measures may provide information about targets of interventions, progress toward behavior goals, and the risk of future outbreaks [3-9]. Although real-time reports on pandemic-related tests and mortality are widely available, there are fewer opportunities to gain near real-time insight into behaviors and beliefs about the pandemic.

Social media, which people are using now more than ever to communicate, has served as a useful data source for providing rapid insight into the public’s behaviors and beliefs during the pandemic [10-13]. Studies have noted a high prevalence of COVID-19–related discussion—including such topics as hygiene, shortages, and the spread of misinformation—and an increase in COVID-19–related discussion as COVID-19 cases increase [5,14,15]. However, existing findings are based on evidence during only the beginning of the outbreak, from December 2019 to April 2020, and the range of topics and keywords explored is also limited [7,14-19]. Additionally, studies analyzing COVID-19 behaviors and beliefs on social media have primarily used Twitter as their source, which has several limitations [14-16,19]. Most notably, highly rated retweets are more likely to come from spam and bot accounts, which are also actively posting about COVID-19, and can obscure the targeting of signals from human discussions [20-22]. Further, previous studies each focused on a particular aspect of the pandemic, such as misinformation relating to the pandemic, without comparing the volume of discussion related to multiple aspects to determine the public’s relative focus on particular pandemic-related issues and behaviors. Therefore, there is a need to assess how the public’s current reaction to the pandemic has changed since the early stages, by examining broad online discussion from more diverse sources.

Accordingly, we measured the prevalence of online discussion that included topics in the categories of daily life, which may or may not be related to COVID-19, and COVID-19–related public health, from June through November 2020. We also assessed the correlation between prevalence of discussion topics and US COVID-19 new daily case rates (incidence). In measuring these trends in social media data and the COVID-19 incidence rate in the United States, we sought to elucidate the US public’s engagement with COVID-19–related public health measures, which are crucial to addressing the current pandemic.

## Methods

### Data Sources

The data sample consisted of unstructured, English-language posts from forums, such as Reddit, Facebook public pages, and 4Chan, and comments from news sites (Table S1 in Multimedia Appendix 1) [23]. We defined forums as thread lists or topic-specific pages, and excluded social media sites including Twitter, YouTube, Instagram, and LinkedIn [24]. Signals Analytics, an advanced analytics consulting firm that conducted the analysis, accessed these data sources through a third-party data vendor, NetBase [25,26]. These social media posts were geotagged by NetBase both directly, by using geolocation data from posts, and indirectly, by using author profiles and unique domain codes (such as .uk). All data were deidentified by NetBase before being transferred to Signals Analytics.

In addition to the social data, the study included US COVID-19 case data from the COVID-19 Dashboard by the Center for Systems Science and Engineering at Johns Hopkins University [27]. These data were updated daily using a public application programming interface (API) and included total number of deaths, new daily deaths, total active cases, and daily new cases [28].

No personal identifying information (eg, usernames, emails, or IP addresses) was shared as part of the analysis or reporting process. This study was exempted from Institutional Review Board review by Yale University as it did not engage in research involving human subjects.

### Approach

To determine trends in social media discussion during the COVID-19 pandemic, we collected data posts from all internet sources and applied natural language processing (NLP) algorithms to identify and classify mentions of COVID-19, COVID-19–related public health measures, and daily life topics (Table S2 in Multimedia Appendix 1).

NetBase ran a daily query that we designed based on our project scope on over 300 million online data sources from June 15 to November 15, 2020 (Methods 1 in Multimedia Appendix 1). There were several steps to narrow the sample retrieved from the query to include only posts relevant to our research question (Figure S1 in Multimedia Appendix 1). First, NLP algorithms were run to remove advertisements and pornography-related sites and posts (Methods 2 in Multimedia Appendix 1). Next, a taxonomy of topics was applied (Methods 3 in Multimedia Appendix 1). The posts that did not include discussion of topics from the taxonomy were deleted. Finally, all news articles and blog posts were deleted from the sample, so that the only remaining data posts were from social outlets (forums and comments on news sites).

The taxonomy was comprised of two categories, COVID-19–related public health measures and daily life behaviors, each of which included multiple topics (Methods 4 in Multimedia Appendix 1). COVID-19 mentions was also an individual topic in the taxonomy, independent of either category. Any post that directly mentioned COVID-19 by name or synonym, including slang such as “Miss Rona,” was classified as including a COVID-19 mention (Table S2 in Multimedia Appendix 1). Taxonomy categories and topics were not exclusive, so that a post was classified as belonging to each taxonomy topic and category that it contained mention of (Table S2 in Multimedia Appendix 1).

Once all posts were classified according to the topics in the taxonomy, we measured trends in these topics over time by tracking the total number of posts that included mentions of each taxonomy topic and category. Classifications of topics and categories were not mutually exclusive, so the same post was able to be classified into multiple topics across any category. Trends were visualized by taxonomy category, COVID-19 mentions, and by the most commonly mentioned taxonomy topics. These trends were visualized with the COVID-19 incidence rate in the United States. We chose to correlate the trends in taxonomy topics with trends in the COVID-19 incidence rate rather than the COVID-19 death rate based on previous literature, which found a correlation between trends in online social chatter and COVID-19 incidence [3,5].

This approach allowed us to identify changes in both topics that prior research in the early stage of the outbreak had shown to be prevalent in COVID-19 discussion, and topics from daily life and COVID-19 literature reviews that were not previously known to be found in COVID-19 discussion, but that may have become apparent as COVID-19 cases or current events changed [15,16,29-33]. Additionally, our approach removed redundant posts, limiting the effect of bots and reposts (Methods 3 in Multimedia Appendix 1). The taxonomy classification was validated by calculating positive predictive value and sensitivity (Methods 5 in Multimedia Appendix 1). We also validated the methodology by applying it to US-specific current events and found that the approach revealed an increase in online social discussion when the given current event topic was most relevant (Figure S2 in Multimedia Appendix 1). This methodology was shown to reveal insights into outbreak characterization and event prediction for the e-cigarette or vaping use–associated lung injury outbreak [34].

## Results

The final data sample consisted of 9,065,733 online social posts that mentioned at least one of the topics in our taxonomy from June 15 to November 15, 2020 (Table 1). The majority (87%) of posts in our sample came from sources that were categorized as forums, including Reddit, Facebook, and 4Chan (Table 2; Table S1 in Multimedia Appendix 1) [23]. The minority of posts (13%) in our sample were derived from comment sections on news sites, including The Hill, a media source focused on politics and business, and Breitbart, a right-leaning media source (Table 2; Table S1 in Multimedia Appendix 1) [35,36]. Most posts in the sample were not able to be directly geotagged due to sources’ data privacy measures and restrictions. A minority were geotagged as from the United States, with the remaining geotagged as from a country other than the United States (Table S3 in Multimedia Appendix 1). Using indirect geotagging provided by NetBase, it was estimated that about 70% of all initial posts collected by the search query were from the United States. In an independent data sample of 100 posts classified by manual review, the algorithm had a positive predictive value of over 80%, which was calculated as the number of posts correctly classified by the taxonomy using NLP algorithms divided by the number of all posts classified by the taxonomy. This was a higher accuracy measure than is found in comparable social media research [30]. Sensitivity was calculated as the number of correct classifications of a topic using the NLP algorithms divided by the total number of posts for the topic identified by manual screening, and we found that our taxonomy approach led to an average classification rate of 92% sensitivity.

Within the data sample, 6,210,255 (69%) posts were classified as including discussion of daily life topics, while 3,390,139 (37%) contained mentions of COVID-19, and 1,836,200 (20%) posts were classified as including discussion of COVID-19–related public health topics (Table 1). The most prevalent topics among the daily life posts were sex life (n=887,457, 14%), food (n=838,513, 14%), and financial concerns (n=710,757, 11%). The most prevalent topic in COVID-19–related public health behaviors posts was wearing face masks (n=1,120,344, 61%), followed by lockdowns (n=457,705, 25%), and social distancing (n=242,105, 13%).

**Figure 1:**
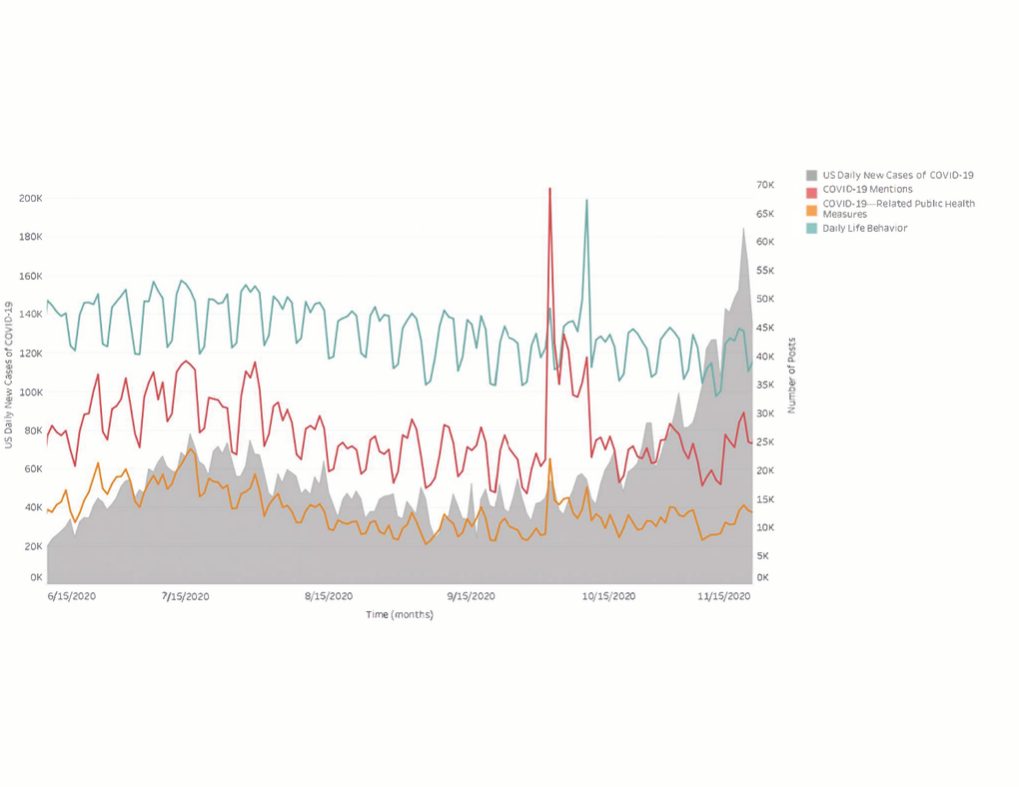
Online social discussion categories versus US daily new COVID-19 cases (June 15 to November 15, 2020).

**Figure 2:**
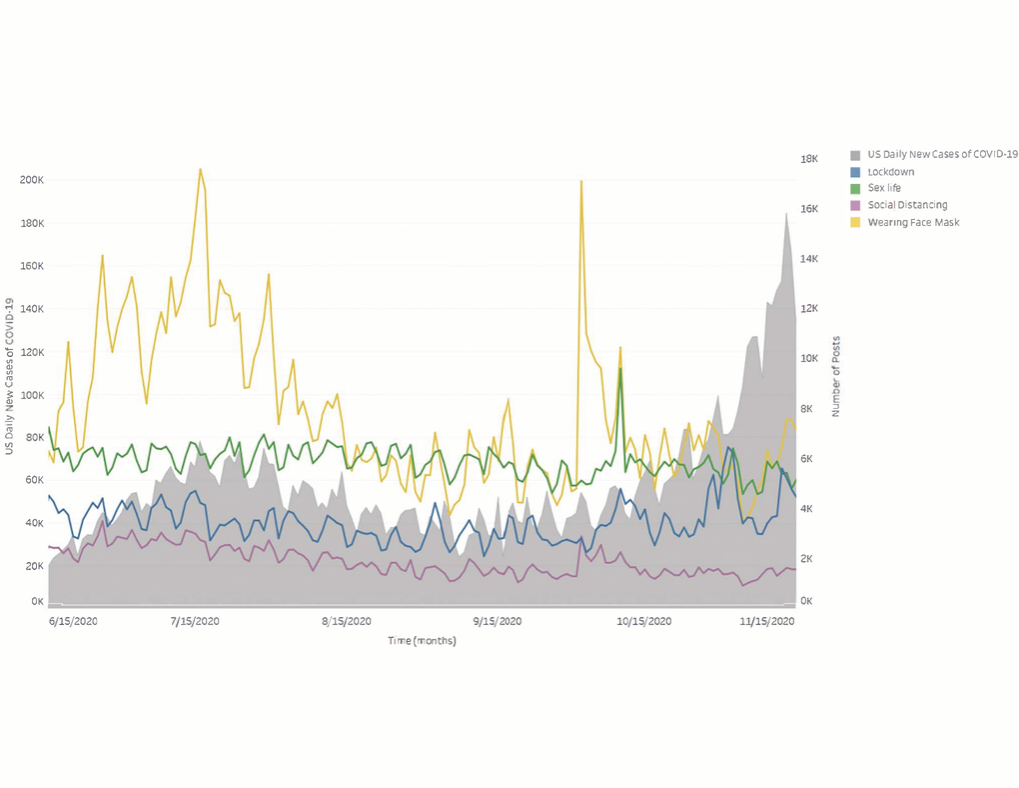
Public health measures online social discussion versus US daily new COVID-19 cases (June 15 to November 15, 2020).

Online social posts including COVID-19 mentions and discussion of COVID-19–related public health behaviors increased in June 2020, as COVID-19 cases also increased, but remained stagnant as cases began to increase in October (Figure 1). Discussion about wearing face masks was most prevalent in mid-July, during the summer wave (mid-June to early September) of COVID-19 cases, and remained at pre-June levels in October and November, with the exception of a sharp increase on October 2, 2020 (Figure 2).

**Table 1:** Number of posts by taxonomy topic from June 15 to November 15, 2020 (N=9,065,733)^a^.

Relevant taxonomy categories (percent classified within all posts) and topics		Number of posts with mentions (percent classified within category)
**COVID-19–related public health topics (20)**		1,836,200
	Wearing face mask	1,120,344 (61)
	Lockdown	457,705 (25)
	Social distancing	242,105 (13)
	Quarantine	94,301 (5)
	Testing	87,712 (5)
	Excessive handwashing	64,679 (4)
	Contact tracing	31,775 (2)
	Reopening	16,681 (1)
	Screening	14,569 (1)
	Wearing gloves	11,531 (1)
	Disinfection	11,076 (1)
	Wearing face shield	10,104 (1)
**Daily life taxonomy topics (69)**		6,210,255
	Sex life	887,457 (14)
	Food	838,513 (14)
	Financial	710,757 (11)
	Travel	651,426 (10)
	Smoking/vaping	476,468 (8)
	Mass gatherings	451,815 (7)
	Virtual communication	414,549 (7)
	Alcohol consumption	398,229 (6)
	Religion	285,538 (5)
	New skills/hobbies acquisition/DIY	280,155 (5)
	Drug use	257,819 (4)
	News/media consumption	257,415 (4)
	Reading	246,074 (4)
	Physical activity	205,116 (3)
	Work from home	198,057 (3)
	Socializing in person	177,522 (3)
	Stockpiling	171,421 (3)
	Relaxation techniques	164,262 (3)
	Excess sleep	127,623 (2)
	Pets	109,510 (2)
	Postponing plans	98,626 (2)
	Childcare	97,735 (2)
	Public transportation	94,414 (2)
	Reduced sleep quality	88,196 (1)
	Home school	80,153 (1)
	Non–COVID-19 hospital visits	77,278 (1)
	Doctor well visit	72,235 (1)
	Funerals	45,394 (1)
	Family-centered time	28,106 (0)
	Outdoor culture	21,546 (0)
	Births	17,283 (0)
	Telehealth	11,574 (0)
	Smokeless tobacco consumption	2136 (0)
COVID-19 mentions (37)		3,390,139

^a^Percentages do not sum to 100 because each post may have included discussion of multiple topics, including topics in different categories.

**Table 2:** Number of posts by source type from June 15 to November 15, 2020.

Category of COVID-19 discussion topic	Posts with mentions of COVID-19–related public health behavior (N=1,836,200), n (%)	Posts with mentions of daily life (N=6,210,255), n (%)	Posts with mentions of COVID-19 (N=3,390,139), n (%)	Total data sample (N=9,065,733), n (%)
Forums	1,494,401 (81)	5,714,446 (92)	2,749,451 (81)	7,928,599 (87)
Comments	341,799 (19)	495,809 (8)	640,688 (19)	1,137,134 (13)

## Discussion

### Principal Findings

Our study had several important findings. From June to November 2020, predominantly US-based online social chatter was more focused on daily life than it was on public health behaviors relating to COVID-19. In addition, although discussion relating to COVID-19 and related public health behaviors appeared to increase with rising US cases in the summer wave (early June to early September), the volume of COVID-19–related discussion was lower in the wave that began in the fall (mid-October), despite the fact that, during the fall wave, COVID-19 cases increased to their highest rates since the pandemic began [37]. In particular, discussion of wearing face masks, the most prevalent of any COVID-19 public health behavior we studied, declined in mid-July despite the pandemic continuing and evidence that wearing face masks has not been universally adopted in the United States, and increased only minimally once cases began to increase again in early October [38,39]. One exception to this finding was the brief but stark increase in COVID-19–related discussion on October 2, 2020, which coincided with the announcement that President Donald Trump had contracted COVID-19 [40]. Our finding that daily life topics were more prevalent in social media chatter than COVID-19–related public health behaviors and mentions of COVID-19 is not immediately surprising given the differences in scope. Nevertheless, we applied consistent methods over time, and the decrease of COVID-19–related discussion in the context of the fall rise in COVID-19 cases differs from the pattern we visualized in the summer wave.

Our study expanded upon previous COVID-19–related social media analyses in that our sources used forums and comments on news sites instead of Twitter and our study was conducted in later phases of the pandemic. Our study sources included forums and comments on news sites, which we believe was an advantage for a few reasons. First, forums are unique to other forms of social media in that they tend to include more text, with greater character allowances and less frequent use of hashtags. This allows the NLP algorithms to be more accurately applied, because forum users include more context to which inclusion and exclusion criteria can be applied. Reddit has also been found to include more discussion than links to external sources, again providing more context to analyze [41]. Second, forums, such as Reddit and public Facebook pages, and comments on news sites, are already focused on specific topics and therefore have more in-depth discussions on the same topic, as opposed to other social media sites, which more often share updates from individual users or links to other sites. The added context from in-depth discussions also allows for more accurate NLP classification. Third, as discussed earlier, retweets driven by spam and bot accounts on Twitter can obscure the targeting of signals from human discussion [20-22].

Due to these differences in study design and time period, our findings may not be consistent with those of previous studies from the first wave of the COVID-19 pandemic. However, future research may investigate whether the cause of the different findings is a significant difference between the type of social chatter found on forums and that found on Twitter and other social media platforms, or whether the different findings are due to a temporal trend of a decreased focus on COVID-19. Although we found that online social chatter was more focused on daily life than it was on COVID-19 public health behaviors, previous research found the opposite. For instance, one study from March 2020 that used data from Twitter found that social media discussion about COVID-19–related health topics was more common than discussion about daily life topics such as socializing, the economy, or politics [42]. Earlier research also found that COVID-19–related public health measures were discussed not only more often than social topics, but also more often than other COVID-19–related topics [7,15]. Thus, our finding that online social chatter from June to November was more focused on daily life than it was on COVID-19 public health behaviors may indicate that the public’s focus on COVID-19 preventative health behaviors had decreased since previous studies were conducted in March and April, or our results may have differed from these earlier studies because our study used different data sources and excluded Twitter. There have been related studies that have analyzed social media data on Reddit—a major source of data in our analysis—during the pandemic; however, none of these studies addressed our research question directly, which was how levels of COVID-19–related public health discussion compared to levels of daily life and COVID-19–related discussion over time. We noted three studies conducted during the time period from January to May 2020 discovered and measured common COVID-19–related topics among online Reddit posts without determining the relative prevalence of COVID-19–related public health discussion to daily life discussion [43-45]. One additional study found that, from February to May 2020, there was a positive correlation between COVID-19–related news coverage and COVID-19–related discussion on the r/Coronavirus subreddit, but that the COVID-19–related discussion declined after sustained media coverage, showing that public attention saturates [46].

Although our results cannot be compared to previous studies to show that public perception changed from the spring wave to the summer and fall waves, there is precedent for the interpretation that the public’s focus on COVID-19 public health measures waned during the fall months. As public health experts warned against relaxing preventive behaviors as pandemic fatigue grew, activity and traffic data indicated that people may have stopped adhering to public health recommendations to stay home and avoid close contact with people outside their household [47-50]. The decline of chatter regarding wearing face masks, and the relative low rates of discussions on other COVID-19–related public health behaviors, may reflect that social media engagement with these issues decreased as the pandemic progressed, and remained low among the US population as the pandemic continued to confront a high COVID-19 daily case rate.

Our study has several limitations. First, although our third-party data provider, NetBase, reported that about 70% of posts were from the United States based on indirect geotagging methods, we do not know the location for most posts according to our direct geotagging methods, which were only able to tag about 20% of posts (Table S3 in Multimedia Appendix 1). As a result, we cannot make international comparisons, but our data set is more representative of the United States than of any other country. Second, the number of posts included in our data set was much lower than previous studies, likely due to the types of data sources used, which excluded social media sites such as Twitter in order to exclude noise that might have obscured signals in data, and our methodology, which included removing posts not relevant to our more refined taxonomy. We used a stringent exclusion criterion with a list of prespecified keywords that may also have led to a smaller sample size, but our approach aimed to create a sample with high accuracy levels. Third, we were not able to include sentiment analysis or other content analysis in our study, which is an area for further exploration. Finally, there is no demographic information available from the data posts directly due to privacy considerations and data use agreements. Thus, we cannot determine whether our data sample contains biases due to the demographics of the people who posted. For instance, Reddit, which was the most common forum source for our data sample, has been found to be used by a younger, male audience [51,52].

### Conclusion

In this study of predominantly US-based COVID-19 social media data from June to November 2020, we observed that COVID-19 and relevant public health measures were discussed less than daily life behaviors on social media, and that discussion on wearing face masks decreased throughout the summer and into the fall, while cases increased. These discussion rates may reveal a need for increased public health messaging as the pandemic continues.

## Appendix

Multimedia Appendix 1 Supplementary data.

## Abbreviations

API: application programming interface
NLP: natural language processing
