# Supplementary material for: Engagement With COVID-19 Public Health Measures in the United States: A Cross-sectional Social Media Analysis from June to November 2020
Source: J Med Internet Res. 2021 Jun 21;23(6):e26655. doi: 10.2196/26655 (PMC8218897; doi:10.2196/26655)
Supplement: Multimedia Appendix 1 [file jmir_v23i6e26655_app1.docx]

**Appendix List of Contents**

Methods S1. Developing data query

Methods S2. Excluding advertisements and pornographic posts

Methods S3. Applying the taxonomy (NLP algorithms)

Methods S4. Generating the taxonomy

Methods S5. Validating the taxonomy

Figure S1. Sample Data Sample Processing Flowchart

Figure S2A. “George Floyd” Classified Mentions in Posts (June-July)

Figure S2B. “RBG” Classified Mentions in Posts (September-October)

Table S1. Top 10 Sources for Data Sample by Source Type

Table S2. Original posts classified by NLP algorithms as containing discussion of taxonomy topics^a^

Table S3. Direct Geotagging Results of Data Sample by Taxonomy Category and COVID-19 Mentions

Methods 1. Developing data query

Data were initially captured using a broad query based on the scope of the project that was designed to be inclusive, in order to capture all potentially relevant posts. A list of topics was initially constructed by multiple analysts using categories deemed to be relevant to the scope of the project, such as daily life behaviors and COVID-19-related behaviors. The analysts studied posts from various online sources relating to these categories to add topics to the list. The list included contextual topics, such as increase, decrease, and days of the week, as well as current events topics, including the terms “Ruth Bader Ginsburg” and “George Floyd.” NetBase ran the query based on the list of topics we provided to them, using a contextual-based proprietary approach. The NetBase query approach is based on the list of topics, as well as Boolean combinations, topics, and relations between the topics.

Methods 2. Excluding advertisements and pornographic posts

To exclude advertisements and pornographic posts, we first sampled hundreds of posts retrieved by the query, and manually determined rules to identify advertisements and pornography using combinations of keywords, such as “coupon link,” “Pornhub,” or “click here for your prize.” Finally, we applied these rules to exclude posts using a proprietary NLP algorithm.

Methods 3. Applying the taxonomy (NLP algorithms)

Signals Analytics used its proprietary NLP engines and algorithms to classify relevant mentions of taxonomy topics. Specifically, Signals Analytics used rulebase technology based on a set of curated rules from RegEx and language processing concepts aimed to identify specific keywords within the text.^34^ Rules also included different synonyms which are associated with a particular taxonomy topic. All the rules for a specific topic were grouped together to ensure comprehensive coverage. The rules were used in a variety of ways: to identify the exact match within the text, to identify the near-exact match within the text, to identify the match using stemming, to use proximity operators to define the distance between two or more keywords, and to identify keywords for inclusion (positive classification) and exclusion (negative classification, which overrode the original classification given).

Using proprietary NLP algorithms, we excluded duplicates of posts included in the sample, as defined as posts that contained the same date, title and content. This was used as a way to reduce the number of posts in the sample from bots, which are usually used to immediately produce identical posts. However, we did count reposted material as a separate mention so as to measure how much people are talking about a given topic, whether the topic was mentioned within reposts or original posts.

Methods 4. Generating the taxonomy

The taxonomy of COVID-19-related and daily life topics was created to classify the posts (Table 2). This taxonomy was created based on: (1) a literature review on social and behavioral changes in response to COVID-19 in the US, (2) consultation with clinical experts, and (3) alignment with our research objectives. The literature review was first conducted on June 18, 2020 using PubMed, medRxiv, and Google Scholar including scholarly articles in the areas of clinical research, public health, and sociology to identify topics highly correlated with the public’s response to COVID-19 on social media. A second literature review, conducted on July 16, 2020 and using medRxiv, arXiv, and PubMed, included scholarly articles in the areas of data science and Internet research to identify COVID-19-related topics found to closely correlate with COVID-19 case rates. Clinician members of our team, CC and SM, reviewed the taxonomy to add terms relevant to COVID-19 public health topics, using a consensus technique. The taxonomy was examined and finalized based on our research objectives while excluding those that could have obscured important trends and signals (e.g., topics with redundant information, such as “panic buying” and “stockpiling,” were collapsed).

A multi-step process was in place by the analysts to enhance and validate a list of keywords and rules corresponding to each topic in the taxonomy. Multiple analysts leveraged online dictionaries, medical dictionaries, and thesauruses, for synonyms to each topic listed in the taxonomy. Once a preliminary list of keywords was completed, a manual screening process was performed on several random samples of the data to identify additional keywords, frequent typos, slang terms, and context of words for higher accuracy and precision. The context of words was used to create further inclusion and/or exclusion criteria for keywords to increase the accuracy of topic classification. For instance, the taxonomy topic, “wearing a facemask” included a contextual rule to exclude references to facials and beauty products from being labeled by the taxonomy topic.

Methods 5. Validating the taxonomy

The taxonomy classification was validated in an independent sample of 100 posts by two analysts through manual review. The 100 posts were collected from the overall sample before the taxonomy was applied. A third analyst was included when there was disagreement. The algorithm had a positive predictive value of over 80% on this independent dataset. We calculated the positive predictive value by determining the ratio of true positives to predicted positives, or of posts correctly classified by the NLP algorithms to contain at least one mention of any taxonomy topic to all posts classified by the NLP algorithms to contain at least one mention of any taxonomy topic. The Signals Analytics team performed ongoing quality assessment analyses, completing timely checks of taxonomy topics for accuracy and updating the list of keywords and rules according to the evolving nature of the social media data. A further sensitivity calculation was done by multiple analysts. For 3 separate taxonomy topics, the analysts manually classified 100 random posts, which were later compared with the result after the taxonomy was applied using the NLP algorithms. Sensitivity was calculated as the number of correct classifications of a topic using the NLP algorithms divided by the total number of posts for the topic identified by manual screening, and we found that our taxonomy approach led to an average classification rate of 92% sensitivity.

Figure S1. Sample Data Sample Processing Flowchart

Classification: Applying taxonomy to

sample

Relevancy: Removing ads and pornographic posts from sample

Sourcing: Excluding news and blog data from sample

**Initial query**

Figure S2A. “George Floyd” Classified Mentions in Posts (June-July)

**
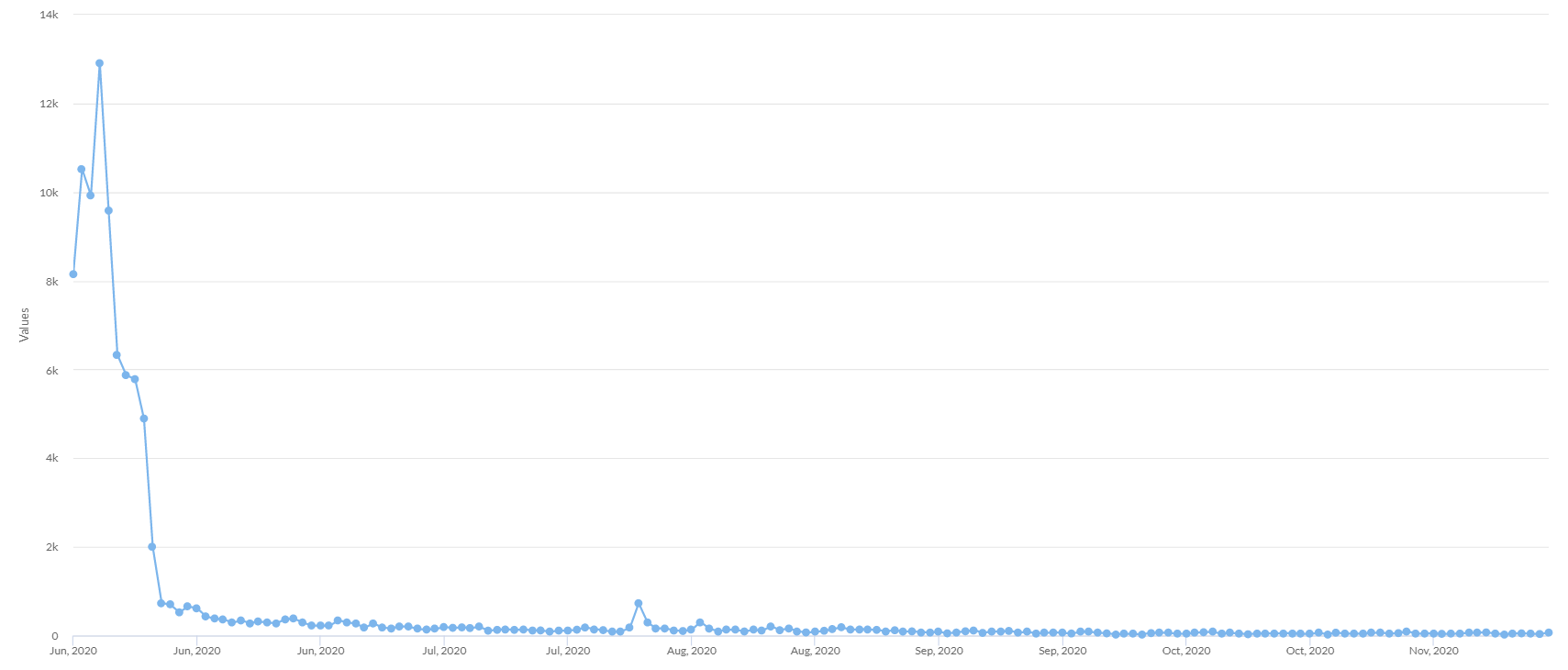
**

Number of Posts Including Topic Mentions

Time (Biweekly)

Figure S2B. “RBG” Classified Mentions in Posts (September–October)

**
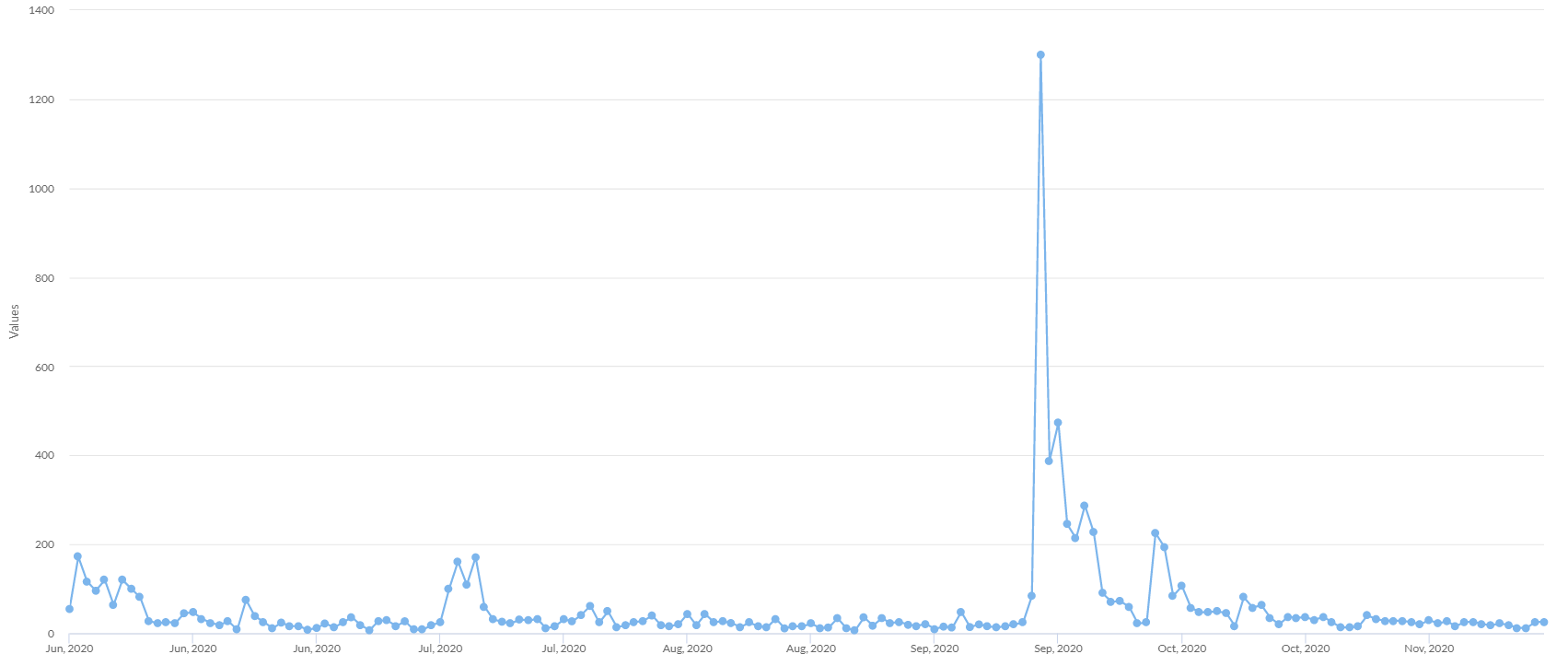
**

Number of Posts Including Topic Mentions

Time (Biweekly)

Table S1. Top 10 sources for data sample by source type^a^

|  | **Forums** | **Comments** |
| --- | --- | --- |
| **1** | www.reddit.com | thehill.com |
| **2** | www.facebook.com | www.breitbart.com |
| **3** | boards.4chan.org | www.mediaite.com |
| **4** | boards.4channel.org | abcnews.go.com |
| **5** | community.whattoexpect.com | www.worldstarhiphop.com |
| **6** | www.mumsnet.com | pjmedia.com |
| **7** | www.tripadvisor.com | townhall.com |
| **8** | www.booking.com | www.thegatewaypundit.com |
| **9** | community.babycenter.com | www.rawstory.com |
| **10** | www.airbnb.com | www.bitchute.com |

^a^Sources are listed in descending order by volume of posts. The number of posts from our sample that was derived from each source was not available to us from NetBase.

Table S2. Original posts classified by NLP algorithms as containing discussion of taxonomy topics^a^

| **Original Post** | **Taxonomy Topic** | **Keywords in Text** |  |
| --- | --- | --- | --- |
| **Need to renew (foreign) passport at a consulate in NYC - Questions about miss rona?** So my parents need to renew their (foreign) passports in a consulate in NYC, in UES.I am going with them as they're new to the country and need help with it. I read the travel advisory and regulations on the department of health website. We are flying in from MI.I know we are supposed to fill in a (contact tracing) form while on the plane before we land. If we don't have that, we can be fined up to $2000 and there are usually people at the gates that check for those. I also know we are supposed to quarantine for 14 days but we're gonna be there for 1.5 days. We were wondering if we would be ok with visiting open-spaces like Central Park while wearing masks and maintaining social distance. Are there usually people walking around checking if we have quarantined or not? Any overall advice about visiting? | COVID-19 | Miss Rona |  |
|  | Wearing Face-Mask | wearing masks |  |
|  | Social Distancing | social distance |  |
|  | Travel | flying in |  |
| **Things to do on a birthday when you cant go out?** Buy the most expensive whiskey you can affort. Lagavulin 18 comes to mind. Laproiagh is good as well. Finlaggan, etc. All smokey, tho. You can't go wrong with a good Baileys if you're on the Northern Hemisphere. Food: I would order a few buckets of KFC, so you can eat the leftover then next day. And download/buy the LOTR extended editions or so. and have fun. Another idea is to go onto tinder or so and find a Covid-F buddy. This means you only have intercourse with each other and do not have intercourse with others as long Lockdown/Covid is a thing | COVID-19 | Covid |  |
|  | Sex life | F buddy / intercourse |  |
|  | Food order / Take-away | order *in proximity* KFC |  |
|  | Lockdown | lockdown |  |
| My roommate who complains about being poor and that they make more money on unemployment than their job literally spent all summer ordering shit on amazon. She probably spent every cent of stimulus and unemployment on random shit. It’s really a mindset about wasteful spending that allows Amazon to beat earnings by 10x during a global pandemic. People are stupid with their money and buy stupid shit. | Financial Concerns | being poor |  |
|  | Shopping Online | order *in proximity* amazon |  |
|  | Unemployment | unemployment |  |

^a^All author names and dates were removed.

Table S3. Direct geotagging results of Data sample by taxonomy category and COVID-19 mentions

| Country | Number of Daily Life Posts (%)  N=6,210,255 | Number of COVID-19-Related Public Health Posts (%)  N=1,836,200 | Number of COVID-19 Mentions (%)  N=3,390,139 |
| --- | --- | --- | --- |
| **US** | 1,016,763 (16) | 235,418 (13) | 478,849 (14) |
| **United Kingdom** | 146,594 (2) | 50,862 (3) | 90,065 (3) |
| **Not Mentioned** | 4,888,789 (79) | 1,529,017 (83) | 2,730,332 (81) |
